# μ Opioid Modulation of Sensorimotor Functional Connectivity in Autism: Insights From a Pharmacological Neuroimaging Investigation Using Tianeptine

**DOI:** 10.1016/j.bpsgos.2025.100663

**Published:** 2025-12-04

**Authors:** Mihail Dimitrov, Nichol M.L. Wong, Sydney Leaman, Lucas G.S. França, Ioannis Valasakis, Jason He, David J. Lythgoe, James L. Findon, Robert H. Wichers, Vladimira Stoencheva, Dene M. Robertson, Sarah Blainey, Glynis Ivin, Štefan Holiga, Mark D. Tricklebank, Dafnis Batalle, Declan G.M. Murphy, Gráinne M. McAlonan, Eileen Daly

**Affiliations:** a Department of Forensic and Neurodevelopmental Sciences, Institute of Psychiatry, Psychology and Neuroscience, King’s College London, London, United Kingdom; b Department of Psychology, The Education University of Hong Kong, Hong Kong, China; c Centre for Psychosocial Health, The Education University of Hong Kong, Hong Kong, China; d Medical Research Council (MRC) Centre for Neurodevelopmental Disorders, King’s College London, London, United Kingdom; e Department of Early Life Imaging, School of Biomedical Engineering and Imaging Sciences, King’s College London, London, United Kingdom; f Department of Computer and Information Sciences, Faculty of Engineering and Environment, Northumbria University, Newcastle upon Tyne, United Kingdom; g Department of Biomedical Computing, School of Biomedical Engineering and Imaging Sciences, King’s College London, London, United Kingdom; h Cognitive Neuroscience Unit, School of Psychology, Deakin University, Geelong, Victoria, Australia; i Centre for Social and Early Emotional Development, Deakin University, Geelong, Victoria, Australia; j Department of Neuroimaging, Institute of Psychiatry, Psychology and Neuroscience, King’s College London, London, United Kingdom; k Department of Psychology, School of Mental Health and Psychological Sciences, Institute of Psychiatry, Psychology and Neuroscience, King’s College London, London, United Kingdom; l N=You Neurodevelopmental Precision Center, Department of Child and Adolescent Psychiatry and Psychosocial Care, Division of Woman-Child, Amsterdam UMC, Amsterdam, the Netherlands; m Adult ADHD and Autism Service, Croydon Adult and Behavioural and Developmental Psychiatry Directorate, South London and Maudsley NHS Foundation Trust, London, United Kingdom; n South London and Maudsley NHS Foundation Trust Pharmacy, London, United Kingdom; o Roche Pharma Research and Early Development, Roche Innovation Center Basel, F. Hoffmann–La Roche Ltd., Basel, Switzerland; p NIHR Maudsley Biomedical Research Centre at South London and Maudsley NHS Foundation Trust and the Institute of Psychiatry, Psychology and Neuroscience, King’s College London, London, United Kingdom

**Keywords:** μ opioid, Autism, Degree centrality, Functional connectivity, rs-fMRI, Tianeptine

## Abstract

**Background:**

Reproducible patterns of atypical functional connectivity (FC) of sensorimotor and higher-order networks have previously been identified in the autistic brain. However, the neurosignaling pathways underpinning these differences remain unclear. The μ opioid system is involved in sensory processing as well as social and reward behaviors and has been implicated in autism, suggesting a potential role in shaping the autistic brain. Therefore, we tested the hypothesis that there is atypical involvement of the μ opioid system in these networks in autism.

**Methods:**

We used a placebo-controlled, double-blind, randomized, crossover study design to compare the effects of a single dose of the μ opioid receptor agonist tianeptine in autistic (*n* = 20) and nonautistic (*n* = 21) males on FC of sensorimotor and frontoparietal networks.

**Results:**

We found that tianeptine increased FC of a sensorimotor network previously characterized by atypically low FC in autism. The connectivity of the frontoparietal network was not significantly shifted.

**Conclusions:**

Our findings suggest that μ opioid neurosignaling may contribute to functional brain differences in the sensorimotor network in autism. Given that sensorimotor system alterations are thought to be central to autism and contribute to other core autistic features, as well as adaptability and mental health, further research is warranted to explore the translational potential of μ opioid modulation in autism.

## Plain Language Summary

Reproducible patterns of atypical brain connectivity have been identified in autism. However, the underlying molecular mechanisms remain unclear. In this study, we measured the effects of the μ opioid receptor agonist tianeptine on brain connectivity in autistic and nonautistic men. We found that, in the former, tianeptine increased connectivity of sensorimotor regions previously characterized by atypically low connectivity. This suggests a role for μ opioid signaling in sensorimotor differences in autism and possibly downstream adaptive behaviors and mental health.

Autism is a heterogeneous spectrum of neurodevelopmental conditions that is characterized by social communication differences and repetitive and stereotyped behaviors, including sensory atypicalities (1). It is present in up to 2.8% of the population (1, 2, 3, 4) and is associated with an increased likelihood of physical and mental health conditions (such as depression and anxiety), higher rates of unemployment, and lower overall well-being and quality of life (1). Nevertheless, pharmacological intervention options for those who would like that choice are still largely unavailable (1,5), as are validated and meaningful stratification and candidate drug response biomarkers (6). This may be due in part to our incomplete understanding of autism neurobiology.

Multiple previous hypotheses have been proposed that the neurobiological underpinnings of autistic behaviors pivot around differences in brain connectivity. Recent large-scale resting-state functional magnetic resonance imaging (rs-fMRI) studies have reported subtle but reproducible differences across different datasets in so-called resting-state functional connectivity (rsFC), with overconnectivity of higher-order networks and underconnectivity of sensorimotor networks (7,8). However, the mechanism(s) underpinning this are unclear. Given that synaptic events shape brain connectivity (9,10), one possible explanation is that group differences in rsFC patterns are driven by altered neurosignaling. In support of this suggestion, there is evidence of atypicalities in multiple molecular systems in autism (11,12), hinting at a possible causal relationship. We have previously shown that several of those systems are likely involved in atypical brain function in autistic individuals (13, 14, 15, 16, 17, 18, 19, 20, 21, 22, 23, 24, 25, 26). Nevertheless, the impact of neurosignaling on a key metric of large-scale rsFC in autism has not been directly examined before. Therefore, we used our shiftability approach, described in Whelan *et al.* (27), to test the hypothesis that large-scale rsFC in autism is regulated differently by the μ opioid system. Specifically, we used a single dose of the atypical antidepressant/anxiolytic tianeptine, a μ opioid receptor (MOR) agonist (28, 29, 30), to compare autistic and nonautistic brain responses of functional networks with known reproducible differences in autism (across large samples) as reported by Holiga *et al.* (7).

Tianeptine was selected based on evidence that its MOR target is implicated in autism-relevant processes related to neuronal development and synaptic plasticity (31) as well as to sensory, reward, and social behaviors (12). Furthermore, the MOR system has been specifically linked to autism by several genetic, case, and preclinical studies (12,32, 33, 34, 35, 36, 37, 38, 39, 40, 41, 42, 43, 44, 45, 46, 47). It is worth noting that although safety is prioritized in selecting drugs to target receptor systems in humans and although we interpret the effects of tianeptine as acting on MOR, we acknowledge that tianeptine has non-MOR effects on multiple other systems, including glutamate and GABA (gamma-aminobutyric acid) (29,48).

We measured brainwide rsFC using the same weighted degree centrality (wDC) index as in Holiga *et al.*’s (7) report in order to examine whether the rsFC atypicalities presented in their study may be underpinned by atypicalities in the μ opioid system. wDC is a graph theory measure of node importance in a network. It reflects the sum of all weighted connections between a node and all other nodes (49) across the whole brain (1 voxel = 1 node).

We hypothesized that, as demonstrated in Holiga *et al.* (7), the autism group at baseline would be characterized by hyperconnectivity in higher-order frontoparietal regions and hypoconnectivity in lower-order sensorimotor regions compared with the nonautistic group. However, given the effect sizes reported by Holiga *et al.* (7) (Cohen’s *d* ∼ 0.2–0.64) and given our modest sample size in this demanding pharmacological study (*n* ∼ 20 per group) compared with sample sizes of between 200 and 400 individuals per group in Holiga *et al.*’s work (7), we did not expect a statistically significant difference at baseline. In contrast, as our main focus was on the regulation or responsivity of large-scale networks, we anticipated that 1) tianeptine would cause differential wDC shifts in the autistic and nonautistic groups; 2) tianeptine would shift autistic wDC toward baseline nonautistic group levels., i.e., in the autism group, it would decrease wDC of frontoparietal and increase wDC of sensorimotor networks.

## Methods and Materials

### Study Ethics and Design

This shiftability (27) study was conducted in accordance with the Declaration of Helsinki at the Institute of Psychiatry, Psychology and Neuroscience (IoPPN), King’s College London (KCL) in London, United Kingdom. Our investigation did not address safety or clinical efficacy, and the U.K. Medicines and Health Regulatory Authority confirmed that the study was not a clinical trial of an Investigational Medicinal Product as defined by the EU Directive 2001/20/EC. The protocol was registered on clinicaltrials.gov for transparency (NCT04145076). Ethical approval was received from a U.K. Health Research Authority (Stanmore Ethics Committee, 14/LO/0663).

The study evaluated the effects of a single dose of tianeptine at maximum plasma concentration on rs-fMRI compared with inactive placebo in a sample of autistic and nonautistic men.

All participants gave written, informed consent after receiving a complete description of the study. Each person completed 2 rs-fMRI scan sessions following administration of either a single 12.5 mg dose of encapsulated tianeptine (Servier Laboratories) or a single dose of encapsulated placebo (ascorbic acid) in a randomized, double-blind, crossover design. The 12.5-mg dose was selected as this a standard dose used in clinical practice (although 3 times a day). The randomization list for the administration order was produced using a computerized random number generator with block randomization. Scanning commenced approximately 1 hour after the participant received a dose as tianeptine reaches peak plasma levels 0.94 hour (±0.47 hour) following administration (50). There was a minimum interscan interval of 8 days to allow for complete drug washout (t_1/2_ = 2.5 hours [±1.1 hours] (washout = 10 × t_1/2_ = 25 hours) (50). Each participant was examined by a medical doctor prior and subsequent to administration of both doses.

### Participants

Participants were recruited via several channels: existing study databases (only if consent for contact was available); South London and Maudsley services; advertisements on the university website, at autism events (e.g., The Autism Show), and through autism groups, including the National Autistic Society and Autistica; and word of mouth.

During the screening phase, participants were excluded if they had an intellectual disability (ID) (IQ < 70), any major mental health condition (e.g., psychosis, bipolar disorder, or major depressive disorder), genetic disorders associated with autism, substance dependence, or if they were taking medication targeting the serotonergic system [at the time of data collection, it was still assumed that tianeptine might work as a serotonin-selective reuptake enhancer; evidence for its MOR action was published in 2014 (28) and 2017 (30)]. Diagnoses of autism spectrum disorder were made by consultant psychiatrists using ICD-10 research criteria (51) and confirmed using the Autism Diagnostic Interview-Revised (52) if an informant was available. Current autistic symptoms were assessed by means of the Autism Diagnostic Observation Schedule (53). IQ was measured using the Wechsler Abbreviated Scale of Intelligence (WASI) (54).

Twenty-one nonautistic men (age range = 18–52 years, mean = 26) and 20 autistic men (age range = 19–50 years, mean = 29 years) were included in the study. Data from 2 nonautistic participants (2 placebo sessions and 2 drug sessions) and 3 autistic participants (2 placebo sessions and 1 drug session) were excluded from the analysis due to significant head movement, resulting in a final sample of 19 nonautistic men and 20 autistic men (*n*_placebo_ = 18, *n*_drug_ = 19). The sample size was based on results from previous shiftability studies [see Wichers *et al.* (23)].

### MRI Data Collection

MRI data were acquired using a 3T GE Discovery MR750 scanner equipped with an 8-channel birdcage head coil at the Centre for Neuroimaging Sciences, IoPPN, KCL, London, United Kingdom.

Structural MRI data were collected using a 3-dimensional inversion recovery prepared fast spoiled gradient recalled (IR-FSPGR) sequence. Specifically, we used the second-generation sequence (55) developed by the MRI core of the Alzheimer’s Disease Neuroimaging Initiative (56) with the following MR parameters: TR = 7.312 ms, TE = 3.016 ms, inversion time = 400 ms, FA = 11°, FOV = 270 mm, matrix size = 256 × 256 voxels, voxel size = 1.055 × 1.055 × 1.2 mm, 196 sagittal slices.

An echo-planar imaging (EPI) sequence with the following MR parameters was used for the acquisition of the rs-fMRI data: TR = 2300 ms, TE = 12.7/31/48 ms, FA = 90°, FOV = 240 mm, matrix size = 64 × 64 voxels, voxel size = 3.75 × 3.75 × 4.2 mm, 33 axial slices (per volume), no. of volumes = 215, length = 8.24 minutes. During the functional scan, participants were asked to keep their eyes open and fixated on a cross.

### MRI Data Preprocessing and Quality Control

The rs-fMRI data were preprocessed using a pipeline nearly identical to the one used by Holiga *et al.* (7). It was implemented in AFNI (version 21.1.07) (57) and consisted of the following steps: removal of non–steady-state volumes, despiking, slice time correction, registration of the rs-fMRI image to the structural image, normalization to standard (i.e., MNI152_T1_2009c) space, tissue segmentation, optimal combination of echoes and motion correction using multi-echo independent component analysis (58,59), smoothing (6 mm^3^), scaling (to percent signal change with μ = 0), regression of white matter and cerebrospinal fluid signal, and bandpass filter (0.01–0.1 Hz) and censoring (if framewise displacement [FD] >3 mm and/or if motion outlier [i.e., >2σ]).

The preprocessed data were then passed through a qualitative check using the quality control (QC) reports that are automatically generated by the AFNI software. Specifically, data were inspected for artifact-free structural and functional images; adequate skull stripping; goodness of fit of the registration and the Montreal Neurological Institute normalization; presence of defined default mode, visual, and auditory networks following seed-based rsFC estimation; subthreshold percentage of censored volumes (arbitrarily set to 20%), and signal coverage similar or superior to the mean coverage.

### FC Estimation and Postprocessing

The preprocessed and QC-cleared rs-fMRI data were further processed to obtain a measure of rsFC, namely the graph theory metric used by Holiga *et al.* (7), i.e., wDC. The wDC index represents the sum of weighted connections of each voxel. Accordingly, wDC was calculated for each voxel (in each participant, for each condition) by taking the sum of all correlation coefficients [computed using cosine similarity, S_C_ (60,61)] between the *z* score–standardized (i.e., μ = 0, σ = 1) time series of that voxel and the rest of the voxels in the gray matter of the brain (see formula below).

(1) $$wDC\left(a\right)=\sum_{i=1}^{n}S_{C}\left(a,b_{i}\right)=\sum_{i=1}^{n}\cos\left(\theta_{i}\right)=\sum_{i=1}^{n}\frac{a\;.\;b_{i}}{\left|\left|a\right|\right|\;.\;\left|\left|b_{i}\right|\right|\;}$$

for a given voxel time series vector *a* and the time series vectors of all other voxels *b*_*i*_ in the gray matter of the brain (for a single participant in a single condition), where θ_i_ is the angle between *a* and *b*_*i*_.

N.B. *a* and *b*_*i*_ are 205-dimensional vectors as there are 205 time points.

Before estimating wDC, the raw correlation matrices were thresholded (S_C_ > 0.25) (62,63), similar to Holiga *et al.* (7), in order to eliminate low temporal correlation as it likely reflects noise.

**Figure 1:**
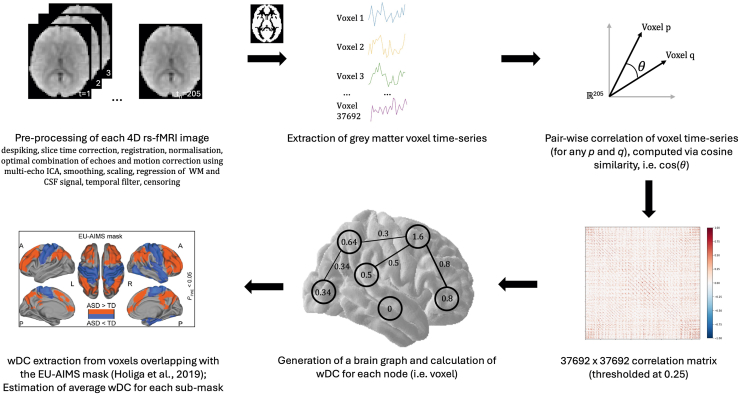
Schematic of the image processing pipeline. EU-AIMS (European Autism Interventions) mask image adapted with permission from Holiga *et al.* (7). 4D, four-dimensional; ASD, autism spectrum disorder; CSF, cerebrospinal fluid; ICA, independent components analysis; rs-fMRI, resting-state functional magnetic resonance imaging; TD, typically developing; wDC, weighted degree centrality; WM, white matter.

The resulting wDC images were *z* scored, hereafter referred to as wDC maps. The maps were then constrained to regions that had been reported as significantly different between autistic and nonautistic individuals by Holiga *et al.* (7). This was achieved by masking out voxels that fall outside of the EU-AIMS (European Autism Interventions) mask from this 2019 study. The masked wDC maps were further split into 2 submaps: one corresponding to a higher-order network of hyperconnected frontoparietal regions (4446 voxels) and another corresponding to a network of hypoconnected sensorimotor regions (2803 voxels). In practice, the EU-AIMS mask was first split into its 2 constituents, and each of the submasks was resampled to the preprocessed rs-fMRI data, binarized, and intersected with the common gray matter mask that was itself generated by intersecting all participant-specific gray matter masks (threshold = 0.85%) using the FMRIB Software Library (FSL) (64). The wDC values comprising each submask were averaged to obtain a mean wDC value for each of the 2 submasks in each participant in each condition. The image processing pipeline is summarized in Figure 1.

### Statistical Analysis

Groups were compared on age, IQ, and in-scanner movement (indexed by mean FD [mFD]). Two IQ scores were missing (1 per group) as 2 participants refused to have the WASI administered to them. Each of these 2 scores was mean imputed using the rest of the scores from the respective group. Normality was confirmed using the Shapiro-Wilk test in addition to a visual inspection of a frequency distribution histogram and a quantile-quantile plot. Equality of variances was assessed using either the Bartlett test or the Levene test (for non-normal distributions). Group comparisons were performed using independent or paired 2-sample Student’s *t* tests (as appropriate), or in the case of non-normally distributed data, via nonparametric alternatives, i.e., Wilcoxon rank-sum or Wilcoxon signed-rank tests, respectively.

The main effects of group and drug as well as the effect of their interaction on wDC were estimated in a linear mixed-effects model (LMM) using restricted maximum likelihood, implemented in R using the *lme4* package. An LMM was fitted for each of the 2 networks of regions: *y* [wDC] ∼ β_0_ + β_1_ [group] + β_2_ [drug] + β_3_ [group × drug] + β_4_ [mFD] + (1 | Participant ID), with (1 | Participant ID) representing a random intercept for each participant to take into account within-subject correlation and individual differences in baseline wDC. Another set of LMMs was fitted to investigate within-group drug effects (2 LMMs for each of the 2 networks): *y* [wDC] ∼ β_0_ + β_1_ [drug] + β_2_ [mFD] + (1 | Participant ID). Permutation testing was applied to establish the statistical significance of each effect in each model (using *p-testR* in R). Although it has been suggested that 1000 permutations are sufficient to produce an accurate approximate permutation test (65), we performed 5000 permutations of the outcome variable (wDC) to obtain more robust estimates in our relatively small sample. Finally, the outputs of each model were corrected for multiple comparisons using the 2-stage Benjamini-Yekutieli false discovery rate (FDR) method (R *stats*) (66) to account for potentially complex dependency structures, including negative correlations (α value = 0.05).

## Results

### Sample Characteristics

Groups did not differ in terms of age or IQ (Table 1). However, the autistic group was characterized by a significantly higher degree of head motion at baseline, as measured by mFD (in mm) (Table 2). Therefore, mFD was included as a covariate in each of the LMMs assessing μ opioid–induced shifts in wDC.

**Table 1:** Demographic and Clinical Characteristics of the Sample

	Neurotypical, *n* = 19	Autistic, *n* = 20	Statistic	*p* Value
Demographics				
Age, Years	26 (7)	29 (10)	*w* = 150.5	*p* = .388
IQ	114 (10)	111 (16)	*t* = 0.659	*p* = .515

Clinical Characteristics				
ADOS				
Communication	–	2 (2)	–	–
Social interaction	–	6 (3)	–	–
Imagination	–	1 (0)	–	–
Repetitive behaviors	–	1 (2)	–	–
ADI-R				
Communication	–	19 (9)	–	–
Social interaction	–	13 (6)	–	–
Repetitive behaviors	–	5 (2)	–	–

Values are presented as mean (SD).

ADI-R, Autism Diagnostic Interview-Revised; ADOS, Autism Diagnostic Observation Schedule.

**Table 2:** Degree of Head Motion in the Sample

Between-Group mFD	Neurotypical	Autistic	Statistic	*p* Value
Baseline	*n* = 19	*n* = 18	*w* = 97	*p* = .026^a^
	0.10 (0.05)	0.15 (0.08)		
Drug	*n* = 19	*n* = 19	*w* = 110	*p* = .106
	0.11 (0.04)	0.13 (0.05)		
Within-Group mFD	Baseline	Drug		

Neurotypical	*n* = 19	*n* = 19	*w* = 71	*p* = .352
	0.10 (0.05)	0.11 (0.04)		
Autistic	*n* = 17	*n* = 17	*w* = 66	*p* = .644
	0.15 (0.08)	0.13 (0.05)		

Values are presented as mean (SD). mFD is estimated in mm.

mFD, mean framewise displacement.

a Indicates statistically significant results with α error at 5%.

### Differential μ Opioid Control of Sensorimotor and Higher-Order Networks in Autistic and Nonautistic Individuals

There was no main effect of group for either of the 2 networks. We observed interaction effects in the hypothesized directions, with a significant sensorimotor shift, which did not, however, survive FDR correction (*t*_34_ = 2.62, *p* = .185). The interaction effect on frontoparietal wDC was not significant (*t*_34_ = −1.72, *p* = .431), suggesting that it might be capturing some of the main effects. Consequently, we ran an additional model without the interaction term and compared the model fits. Both the Akaike information criterion (−32.78 vs. −35.39) and Bayesian information criterion (−16.84 vs. −21.73) favored the simpler model. In both instances, the main effect of group was nonsignificant. Comprehensive LMM output is provided in Table S1.

### Within-Group μ Opioid Shifts

In the nonautistic group, tianeptine did not elicit wDC shifts in frontoparietal or sensorimotor networks. In both sets of regions, individual trajectories were characterized by a pattern in which participants with above-median wDC at baseline tended to undergo positive shifts following drug administration and vice versa.

In the autistic group, tianeptine caused a negative wDC shift in frontoparietal regions, which did not reach statistical significance (*t*_31_ *=* −2.18, *p* = .283). Conversely, the MOR agonist significantly increased sensorimotor wDC (*t*_16_ = 3.72, *p* = .043). The same pattern that was present in the individual trajectories of the nonautistic participants was also noted in the autistic group, although it was limited to frontoparietal areas. In the sensorimotor network, we observed a general increase in wDC, regardless of baseline wDC.

**Figure 2:**
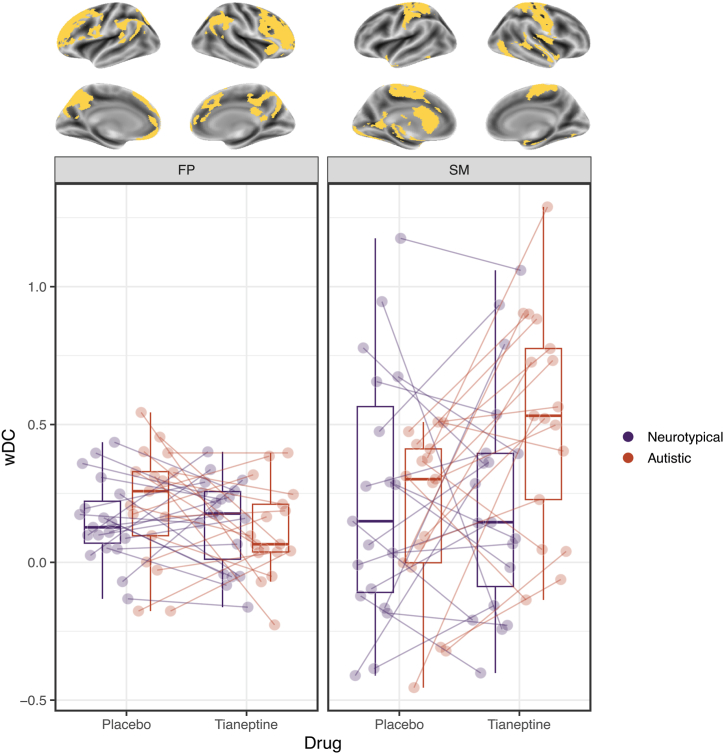
The effect of tianeptine on weighted degree centrality (wDC) averaged across frontoparietal (FP) (left) and sensorimotor (SM) (right) brain regions in neurotypical and autistic individuals. The brain plots (top) show the regions comprising each mask.

Visual representation of the results is shown in Figure 2. Detailed output from the LMMs can be found in Tables S2 and S3.

## Discussion

We found that in the autistic group, administration of the MOR agonist tianeptine shifted sensorimotor wDC, a network measure of node importance. This particular cluster of sensorimotor regions has previously been reported as atypically hypoconnected (indexed by decreased wDC) by Holiga *et al.* (7) using data from several large-scale studies comparing autistic with nonautistic individuals in a broad age range. Here, we showed that MOR modulation can increase wDC in this specific cluster. This suggests that reproducible functional brain atypicalities in sensorimotor regions of the autistic brain that have been observed in large-scale studies may be driven by atypical MOR function.

Differences in sensorimotor processing and behaviors are commonly reported in the autistic population (67, 68, 69) and form part of the diagnostic criteria for autism spectrum disorder in DSM-5 (70) and ICD-11 (71). Unusual sensorimotor features are among the earliest signs of autism (72, 73, 74), persist throughout life (70), and exert possible cascading effects on higher-order social and cognitive characteristics of this neurodevelopmental condition (67, 68, 69,75). In addition, the presence of sensory atypicalities in autistic individuals can negatively affect adaptive behavior (76,77) and has been linked to mental health conditions such as depression (78,79) and anxiety (80, 81, 82), further highlighting the practical importance of elucidating sensorimotor mechanisms in autism.

These behavioral and clinical observations are mirrored in functional brain differences as well. For example, there is evidence pointing to atypical sensorimotor rsFC, reflected by an altered regional homogeneity index, in the brains of neonates with an increased likelihood of autism diagnosis (83). Preterm birth, which has been linked to a greater chance of atypical neurodevelopment (84) including autism (85, 86, 87, 88, 89, 90), has also been associated with an atypical dynamic rsFC profile of a sensorimotor network in the neonatal brain that is predictive of neurodevelopmental and autistic traits at 18 months (91). This atypical sensorimotor rsFC feature appears to be present in the autistic population across a wide age range, as shown by recent large-scale rsFC studies (7,8). Furthermore, it has been reported as the most informative predictor in machine learning–based diagnostic classification (92) and has been linked to differences in sensory processing, social difficulties, and restricted and repetitive behaviors (8). These strands of evidence lend support to the idea that sensorimotor atypicalities in autism can be observed on a behavioral as well as on a biological level, and the latter can not only precede but also potentially predict and explain the former.

However, what remains unclear is how these differences in behavior and underlying gross brain function of sensorimotor systems are themselves underpinned by neurosignaling at a more fundamental level. An underexplored neurosignaling candidate in autism research is the MOR system, as previous studies have mainly focused on GABA, glutamate, and serotonin (5-HT) [for example, (11)]. The MOR plays a role in several interconnected processes, including sensory processing, social behavior, and reward, all of which are known to differ in autism (12). Emerging evidence suggests that the MOR system likely functions atypically in autism (12,32, 33, 34, 35, 36, 37, 38, 39, 40, 41, 42, 43, 44, 45, 46, 47), making it a promising target for investigation. Mechanistically, the MOR is a also plausible molecular modulator of the functional landscape of sensorimotor networks in the brain given its moderate availability in those regions (at least in nonautistic individuals) (93,94). Combined with our results showing that the MOR agonist tianeptine can increase sensorimotor wDC [previously shown to be decreased in autistic individuals (7)], this suggests that the MOR system may contribute to characteristics and behaviors associated with autism. Notably, even if this influence is confined to sensorimotor regions and in turn, sensorimotor behaviors, knock-on effects on other systems known to be atypical in autism may also be present. These effects may contribute to social and cognitive differences and potentially predispose individuals to conditions such as depression and anxiety, which are also associated with MOR atypicalities (95). Therefore, while preliminary, these findings possess a potential translational value and thus warrant further investigation.

Nevertheless, although there are several lines of evidence that emphasize tianeptine MOR agonist actions (28, 29, 30), there are also reports of various other direct (∂ opioid) (28) and indirect effects on other systems, including GABA (29), glutamate (96, 97, 98, 99), dopamine (29,100,101), 5-HT (102, 103, 104, 105, 106, 107, 108), and BDNF-TrkB (brain-derived neurotrophic factor-tropomyosin receptor kinase B) (109,110). Each of these pathways has been found to be atypical in autism (11,111), raising the question of whether the shifts that we observed in this study can solely be attributed to direct MOR effects or whether they are a consequence of a broader multisystem action. It is possible that the two are inseparable given well-documented interactions between MOR and GABA, glutamate, dopamine, 5-HT (112), and BDNF-TrkB (113).

This brings us to another consideration: To date, most drugs that have been tested in autistic individuals to address core and/or co-occurring difficulties have primarily targeted single neurosignaling systems (114). However, autism is a complex condition linked to atypicalities across multiple neural systems (11) as well as in the overall excitation/inhibition balance (115,116) in the brain. Therefore, it is possible that the current dearth of effective and targeted pharmacological approaches in the context of autism (5,114) may partly stem from a mismatch between the complexity of behavioral phenotypes and compounds targeting them.

Despite the putative multipathway effects of tianeptine, its primary action is most likely via the MOR, providing a strong indication that this receptor system may be uniquely positioned to modulate sensorimotor functionality in the human brain, with possible practical implications for autism specifically. Further research is needed to corroborate this and establish whether it has any translational value.

### Limitations

Our findings should be interpreted alongside several key limitations. The analysis was based on a modest sample size dictated by stringent inclusion and exclusion criteria as well as stringent motion correction criteria. This reduced our statistical power, especially with regard to detecting baseline differences between autistic and nonautistic individuals, which (unlike the single-use effects of a psychotropic drug such as tianeptine) are likely to be relatively small (117). However, the repeated-measures design of the study improved power to detect drug effects by decreasing the heterogeneity of the sample. Larger samples may provide more accurate estimates of baseline group differences as well as of tianeptine’s effects on the human brain. Our investigation was limited to adult males with above-average IQ. While this provides an opportunity to probe neurosignaling mechanisms in a more homogeneous sample, it reduces the generalizability of our findings. This has several possible implications. For example, although the adult brain is characterized by a certain level of plasticity, it is nonetheless a system with established homeostatic properties (118). Therefore, it may be less susceptible to external influences and thus may respond differently to pharmacological perturbations compared with the child or adolescent brain. Additionally, previous research has highlighted sex differences in brain structure, function, and connectivity in the autistic population (119, 120, 121, 122, 123) as well as sex differences in pharmacological responses in neurotypical individuals (124). This implies that results obtained in autistic males may not directly generalize to autistic females. Furthermore, our sample consisted of people with above-average intelligence, raising concerns about relevance to individuals with ID, especially in light of the high prevalence of ID in the autistic population (4). Therefore, it is important for future studies to extend our work to include other autistic subgroups. Finally, this study used a single 12.5 mg dose of tianeptine. However, a single dose may not produce effects comparable to those of a sustained administration regimen (e.g., the standard 12.5 mg three times a day used in clinical practice), be it in terms of extent, magnitude, or direction. In addition, evidence suggests that the levels of certain molecular targets of tianeptine may vary seasonally (125,126), which could introduce potential bias in a cross-sectional drug study design such as ours. Further research is needed to address these unresolved questions.

### Conclusions

In this study, we demonstrated that the atypical μ opioid agonist tianeptine increases the centrality of sensorimotor regions previously identified as having reduced centrality in autism at baseline. Given the impact of sensorimotor atypicalities on other core features of autism, adaptability, and mental health, our findings highlight the potential relevance of the μ opioid system for further investigation. While our results suggest that μ opioid neurosignaling may underlie atypical sensorimotor function in autism, whether these findings have practical implications for developing more targeted interventions remains an open question for future translational research.

## Supplementary Material

Tables S1–S3

Key Resources Table
