# Supplementary material for: μ Opioid Modulation of Sensorimotor Functional Connectivity in Autism: Insights From a Pharmacological Neuroimaging Investigation Using Tianeptine
Source: Biol Psychiatry Glob Open Sci. 2025 Dec 4;6(2):100663. doi: 10.1016/j.bpsgos.2025.100663 (PMC12860718; doi:10.1016/j.bpsgos.2025.100663)
Supplement: Tables S1–S3 [file mmc1.pdf]

SUPPLEMENTARY INFORMATION

μ-Opioid Modulation of Sensorimotor Functional Connectivity in Autism: Insights From a Pharmacological Neuroimaging Investigation Using Tianeptine

Dimitrov *et al.*

Supplementary materials

Supplementary Table 1. *Results from the interaction analysis. FDR correction has only been applied to effects of interest. Statistically significant effects are highlighted in bold.*

term	estimate	t-statistic	p-value (perm)	p-value (FDR)
**Fronto-parietal**				
(Intercept)	0.27	5.35	0.006	**-**
drug	<0.01	0.08	0.937	-
group	0.11	2.04	0.043	0.283
group X drug	-0.12	-1.72	0.099	0.431
mFD	-1.17	-3.43	**<0.001**	**-**
**Sensorimotor**				
(Intercept)	0.06	0.52	0.972	-
drug	-0.01	-0.14	0.895	-
group	-0.13	-0.98	0.335	1.000
group X drug	0.38	2.62	**0.017**	0.185
mFD	1.69	2.03	**0.042**	**-**

Supplementary Table 2. *The effect of tianeptine on wDC in the non-autistic group. FDR correction has only been applied to effects of interest.*

term	estimate	t-statistic	p-value (perm)	p-value (FDR)
**Fronto-parietal**				
(Intercept)	0.21	2.86	0.243	-
drug	<0.01	0.03	0.980	1.000
mFD	-0.55	-0.89	0.361	-
**Sensorimotor**				
(Intercept)	-0.01	-0.05	0.993	-
drug	-0.02	-0.16	0.872	1.000
mFD	2.39	1.42	0.150	-

Supplementary Table 3*. The effect of tianeptine on wDC in the autistic group. FDR correction has only been applied to effects of interest. Statistically significant effects are highlighted in bold.*

term	estimate	t-statistic	p-value (perm)	p-value (FDR)
**Fronto-parietal**				
(Intercept)	0.43	6.06	0.000	-
drug	-0.12	-2.18	0.052	0.283
mFD	-1.51	-3.70	**0.001**	-
**Sensorimotor**				
(Intercept)	-0.03	-0.17	0.989	-
drug	0.36	3.72	**0.002**	**0.043**
mFD	1.39	1.57	0.148	-
